# Peking Prognostic Score, Based on Preoperative Sarcopenia Status, Is a Novel Prognostic Factor in Patients With Gastric Cancer

**DOI:** 10.3389/fnut.2022.910271

**Published:** 2022-06-06

**Authors:** Jianping Xiong, Haitao Hu, Wenzhe Kang, Yang Li, Peng Jin, Xinxin Shao, Weikun Li, Yantao Tian

**Affiliations:** Department of Pancreatic and Gastric Surgery, National Cancer Center/National Clinical Research Center for Cancer/Cancer Hospital, Chinese Academy of Medical Sciences and Peking Union Medical College, Beijing, China

**Keywords:** gastric cancer, Peking prognostic score, sarcopenia, lymphocyte-to-C-reactive protein ratio, prognostic factors

## Abstract

**Background:**

This study focused on assessing the role of the Peking prognostic score (PPS), a novel prognostic index based on muscle atrophy and lymphocyte-to-C-reactive protein ratio, within gastric cancer patient prognosis.

**Methods:**

We analyzed the data collected from 774 gastric cancer cases between April 2011 and February 2016 (discovery cohort). The results were assessed in 575 gastric cancer cases from March 2016 to September 2019 (validation cohort). For evaluating skeletal muscle mass, we obtained computed tomography images at the third lumbar vertebra level (L3). We performed a time-dependent receiver operating characteristic curve (t-ROC) to analyze PPS’s prognostic significance with others.

**Results:**

The discovery cohort enrolled altogether 774 patients with non-metastatic gastric cancer, including 639 (82.5%) men along with 135 (17.5%) women. The patients were divided into 3 groups; 166 patients (21.4%) were assigned into group 0, 472 (60.9%) in group 1, and 136 (17.7%) in group 2, respectively. An increased PPS was in direct proportion to an elder age, reduced body mass index, higher Pathological Tumor Lymph Node Metastasis stage, perineural invasion, and vascular invasion. We identified PPS to independently estimate patient overall survival (OS) together with disease-free survival (DFS; both *P* < 0.001). Additionally, as revealed by t-ROC analysis, PPS exhibited the highest sensitivity compared with other prognostic scoring systems in predicting patient survival. Finally, we evaluated the prognostic value of PPS in the validation cohort and confirmed that preoperative PPS independently estimates patient OS and DFS.

**Conclusion:**

The PPS accounts for an efficient nutrition-inflammation prognostic scoring system in gastric cancer patients.

## Introduction

Gastric cancer (GC) ranks the 5th place among cancers in terms of its morbidity, and 1,089,103 patients are being diagnosed annually. Also, GC is the 3rd most common reason for cancer-associated mortality, which causes about 768,793 deaths every year (1). Although great achievements have been made in diagnosing and treating GC, more than one half of patients with GC are diagnosed at the late stage; besides, disease relapse is still an important factor associated with the dismal GC survival. For improving the overall survival (OS) for GC cases, the strong prognostic factor that predicts tumor relapse contributes to identifying high-risk cases, conducting follow-up, and deciding the suitable therapeutic strategy postoperatively. Sarcopenia, which is a kind of age-associated losses of muscle strength, mass, and function, has become a serious medical issue in aging societies (2). Sarcopenia is significantly related to non-alcoholic fatty liver disease, liver cirrhosis, or cardiovascular events (3–5). It is recognized that sarcopenia plays a more and more important role in cancer, since low muscularity represents an important predicting factor for dismal survival of different tumors (6, 7). Sarcopenia is frequently seen among patients with GC, with a prevalence of over 7–70% (8). It is markedly related to the dismal long-run prognostic outcome in GC cases undergoing surgical resection (9). Although the exact mechanism is not completely illustrated, some potential candidates are proposed, especially its relationship with inflammation (10).

Cancer-related inflammation is currently regarded as the 7th cancer hallmark, which participates in carcinogenesis and tumor progression of diverse cancers. The baseline serum contents of inflammatory biomarkers, like C-reactive protein (CRP)-albumin ratio (CAR), neutrophil-to-lymphocyte ratio (NLR), lymphocyte-to-monocyte ratio (LMR), and platelet-to-lymphocyte ratio (PLR), are associated with cancer development and prognosis, like GC, colorectal cancer (CRC), and esophageal cancer (EC; 11–13). As discovered by Okugawa et al. recently, the lymphocyte-CRP ratio (LCR) served as the prognostic factor in CRC cases (14). A recent study further confirmed that low LCR served as the factor to independently predict OS and disease-free survival (DFS) of GC cases who underwent gastrectomy (15). LCR was the most accurate indicator to predict patient prognosis relative to those inflammation-based scores like CAR, PLR, and NLR among patients with CRC and GC (14). The prognostic nutritional index (PNI), controlling nutritional status (CONUT) score, modified systemic inflammation score (mSIS), and modified Glasgow prognostic score (mGPS) have been suggested as the useful scoring systems to predict GC prognosis (16–19). A recent study proposed a novel prognostic score, namely, Naples prognostic score (NPS), as the potent prognostic biomarker for CRC (20). However, none of these prognostic scoring systems include preoperative sarcopenia status and LCR. As sarcopenia and LCR can significantly affect GC outcomes, it is speculated that the novel scoring system, namely, Peking prognostic score (PPS), constructed according to the preoperative sarcopenia status and LCR, may provide the optimal prognostic value in patients with GC. In this study, we determined PPS’s effect on predicting GC survival and explored the relation of PPS with additional clinicopathological characteristics. Furthermore, we performed a time-dependent receiver operating characteristic (t-ROC) curve to analyze PPS’ prognostic significance with others. The results were assessed in the validation cohort.

## Materials and Methods

The Institutional Review Board of National Cancer Center/Cancer Hospital, Chinese Academy of Medical Sciences and Peking Union Medical College approved our study (NCC-2497). The need to obtain informed consent from the participants was waived by the Institutional Review Board due to the retrospective nature of the study. This study was performed following the Declaration of Helsinki and the Transparent Reporting of a Multivariable Prediction Model for Individual Prognosis or Diagnosis (TRIPOD) reporting guideline.

### Study Design and Population

**FIGURE 1:**
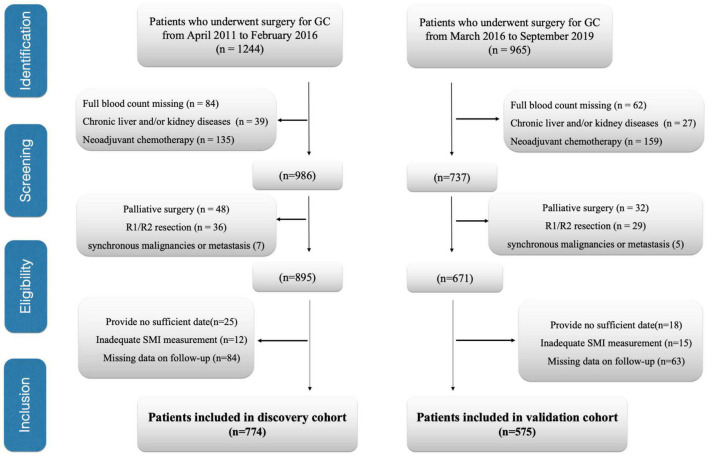
Flow diagram of patients. GC, gastric cancer; SMI, skeletal muscle index.

This study evaluated all cases receiving curative surgical treatment for GC at the Department of Pancreatic and Gastric Surgery at the National Cancer Center/Cancer Hospital, Chinese Academy of Medical Sciences and Peking Union Medical College from April 2011 to February 2016 (discovery cohort). The data presented in Tables 1–3 were collected from the discovery cohort. Patients included in the National Cancer Center/Cancer Hospital from March 2016 to September 2019 were regarded as the validation cohort. The data presented in Supplementary Tables 2–4 were collected from the validation cohort. Patients conforming to the criteria below were excluded: (1) patients undergoing palliative surgical treatment, (2) patients with no routine blood examination preoperatively, (3) patients developing distant metastasis when they received surgical treatment, (4) patients who received neoadjuvant chemotherapy (NACT), (5) patients developing cancers within the remaining organs and/or other synchronous malignancies, (6) those receiving R1/R2 resection, (7) those with insufficient/inexact medical records, (8) patients developing chronic kidney/liver disorders, (9) those with inadequate skeletal muscle index (SMI) measurement, such as edema on preoperative abdominal computed tomography (CT) affects SMI measurements, and (10) the patients having insufficient or unavailable follow-up data. Finally, we enrolled 774 cases in the discovery cohort and 575 cases in the validation cohort (Figure 1). We analyzed subject demographic, histopathological, and laboratory data and later extracted related information from patient records and our hospital database. We determined the clinical tumor stage according to Pathological Tumor Lymph Node Metastasis (pTNM) System (8th edition) formulated *via* American Joint Committee on Cancer. Postoperative follow-up visits were conducted at 3-month intervals in the initial 2 years postoperatively and every 6 months since then. We conducted the final follow-up visit in April 2021. In follow-up visits, we examined tumor markers (CA19-9, CEA, CA72-4), annual endoscopy, abdominopelvic CT, and chest X-ray. This study defined OS as the duration between surgery date and final follow-up or all-cause mortality, which served as a primary end point, and defined DFS as the duration between surgery date and relapse or mortality, which served as a secondary end point. We recorded all-cause mortality as an event.

**TABLE 1:** Association of Peking prognostic score and clinicopathological characteristics in patients with gastric cancer (discovery cohort).

Clinicopathological features	All cases (*n* = 774)	Group 0 (*n* = 166)	Group 1 (*n* = 472)	Group 2 (*n* = 136)	*P* value
**Age** <65.0 ≥65.0	461 (59.6) 313 (40.4)	117 (70.5) 49 (29.5)	285 (60.4) 187 (39.6)	59 (43.4) 77 (56.6)	<0.001
**Gender** Male Female	639 (82.5) 135 (17.5)	138 (83.6) 28 (16.4)	385 (81.5) 87 (18.5)	116 (85.2) 20 (14.8)	0.779
**BMI (kg/m^2^)** ≥18.5 <18.5	723 (93.4) 51 (6.6)	161 (97.0) 5 (3.0)	441 (93.4) 31 (6.6)	121 (89.0) 15 (11.0)	0.001
**Vascular invasion** Negative Positive	501 (64.8) 273 (35.2)	135 (81.2) 31 (18.6)	300 (63.6) 172 (36.4)	66 (48.6) 70 (51.4)	<0.001
**Perineural invasion** Negative Positive	596 (77.1) 178 (22.9)	141 (82.0) 30 (18.0)	267 (77.8) 105 (22.2)	93 (68.4) 43 (31.6)	0.002
**Tumor location** Upper Middle/Lower	227 (29.3) 547 (70.7)	46 (27.6) 120 (72.4)	139 (29.5) 333 (70.5)	42 (30.1) 94 (69.9)	0.113
**pTNM stage** I II III	157 (20.2) 243 (31.5) 374 (48.3)	66 (39.7) 35 (21.1) 65 (39.2)	81 (17.2) 160 (33.9) 231 (48.9)	10 (7.6) 48 (35.3) 78 (57.1)	<0.001
**Adjuvant chemotherapy** No Yes	296 (38.3) 478 (61.7)	88 (53.1) 78 (46.9)	179 (37.9) 293 (62.1)	29 (21.3) 107 (78.7)	<0.001

*BMI, body mass index.*

**TABLE 2:** Univariate and multivariate analysis of clinicopathological variables in relation to overall survival in patients with gastric cancer (discovery cohort).

Clinicopathological features	Univariate analysis	*P*-value	Multivariate analysis	*P*-value
**Age** <65.0 ⩾65.0	Reference 1.33 (1.07, 2.12)	<0.001	Reference 1.25 (1.03, 1.99)	<0.001
**Gender** Male Female	Reference 0.88 (0.68, 2.46)	0.182		
**BMI (kg/m^2^)** ⩾18.5 <18.5	Reference 2.66 (1.42, 3.63)	<0.001	Reference 2.14 (1.23, 2.87)	<0.001
**Vascular invasion** Negative Positive	Reference 1.88 (1.15, 2.29)	<0.001	Reference 1.67 (1.10, 1.92)	<0.001
**Perineural invasion** Negative Positive	Reference 1.79 (1.29, 2.55)	<0.001	Reference 1.63 (1.14, 2.07)	0.011
**Tumor location** Upper Middle/Lower	Reference 0.78 (0.51, 2.32)	0.230		
**pTNM stage** I II III	Reference 2.35 (1.28, 3.62) 9.47 (4.31, 16.42)	<0.001 <0.001	Reference 1.86 (1.14, 2.25) 6.10 (3.12, 8.98)	<0.001 0.012
**Adjuvant chemotherapy** Yes No	Reference 2.64 (1.84, 4.88)	<0.001	Reference 1.69 (1.42, 2.83)	<0.001
**Sarcopenia** Without With	Reference 2.92 (1.35, 4.79)	<0.001	Reference 1.77 (1.18, 2.05)	<0.001
**Lymphocyte: C-reactive protein ratio** >6,000 ≤6,000	Reference 3.39 (2.52, 5.94)	0.001	Reference 2.62 (1.72, 3.38)	<0.001
**PNI** >45 ≤45	Reference 5.69 (2.23, 8.29)	<0.001	Reference 1.87 (1.24, 2.88)	<0.001
**CONUT** <4 ⩾4	Reference 4.74 (2.31, 6.71)	<0.001	Reference 2.61 (1.48, 3.97)	<0.001
**mSIS** 0 1 2	Reference 2.56 (1.45, 4.23) 4.31 (2.89, 7.25)	<0.001 <0.001	Reference 1.60 (1.29, 3.12) 1.93 (1.44, 3.05)	<0.001 <0.001
**mGPS** 0 1 2	Reference 3.78 (2.33, 5.32) 5.29 (2.75, 9.23)	<0.001 <0.001	Reference 2.35 (1.42, 3.16) 3.18 (1.83, 5.74)	<0.001 <0.001
**NPS** 0 1 2	Reference 2.78 (1.40, 5.12) 4.67 (2.85, 8.81)	<0.001 <0.001	Reference 2.21 (1.27, 3.31) 3.26 (1.68, 5.61)	<0.001 0.007
**PPS** 0 1 2	Reference 4.45 (2.51, 7.89) 9.82 (2.94, 16.81)	<0.001 <0.001	Reference 2.32 (1.37, 4.94) 4.67 (2.12, 8.65)	<0.001 <0.001

*PPS, Peking prognostic score; NPS, Naples prognostic score; mSIS, modified systemic inflammation score; CONUT, controlling nutritional status; PNI, prognostic nutritional index; mGPS, modified Glasgow prognostic score; and BMI, body mass index.*

**TABLE 3:** Univariate and multivariate analysis of clinicopathological variables in relation to disease-free survival in patients with gastric cancer (discovery cohort).

Clinicopathological features	Univariate analysis	*P*-value	Multivariate analysis	*P*-value
**Age** <65.0 ⩾65.0	Reference 1.61 (1.15, 2.42)	<0.001	Reference 1.24 (1.07, 1.71)	<0.001
**Gender** Male Female	Reference 0.86 (0.65, 2.19)	0.223		
**BMI (kg/m^2^)** ⩾18.5 <18.5	Reference 2.81 (1.58, 4.73)	<0.001	Reference 2.03 (1.20, 2.65)	<0.001
**Vascular invasion** Negative Positive	Reference 2.17 (1.42, 4.13)	<0.001	Reference 1.59 (1.13, 1.97)	<0.001
**Perineural invasion** Negative Positive	Reference 1.72 (1.22, 2.72)	<0.001	Reference 1.60 (1.18, 2.11)	0.009
**Tumor location** Upper Middle/Lower	Reference 0.81 (0.55, 2.19)	0.316		
**pTNM stage** I II III	Reference 2.67 (1.61, 4.95) 8.55 (3.62, 15.13)	<0.001 <0.001	Reference 1.76 (1.12, 2.09) 5.25 (2.56, 7.24)	0.010 0.019
**Adjuvant chemotherapy** Yes No	Reference 2.71 (1.82, 4.67)	0.007	Reference 1.65 (1.33, 2.62)	<0.001
**Sarcopenia** Without With	Reference 3.16 (1.89, 5.10)	<0.001	Reference 1.72 (1.16, 2.28)	<0.001
**Lymphocyte: C-reactive protein ratio** >6,000 ≤6,000	Reference 4.28 (2.49, 6.15)	<0.001	Reference 2.51 (1.60, 3.46)	<0.001
**PNI** >45 ≤45	Reference 5.77 (2.64, 8.72)	0.011	Reference 1.93 (0.73, 2.57)	0.106
**CONUT** <4 ⩾4	Reference 4.50 (2.16, 7.38)	0.009	Reference 2.13 (0.55, 3.82)	0.325
**mSIS** 0 1 2	Reference 2.43 (1.41, 4.33) 4.76 (2.62, 7.85)	<0.001 <0.001	Reference 1.68 (1.32, 2.76) 2.04 (1.54, 3.20)	<0.001 <0.001
**mGPS** 0 1 2	Reference 3.51 (2.18, 5.80) 4.43 (2.01, 7.72)	<0.001 <0.001	Reference 1.95 (1.64, 3.73) 2.78 (1.36, 5.01)	<0.001 <0.001
**NPS** 0 1 2	Reference 2.64 (1.42, 5.41) 4.88 (2.72, 8.96)	<0.001 <0.001	Reference 2.13 (1.22, 3.25) 3.19 (1.58, 5.54)	0.006 <0.001
**PPS** 0 1 2	Reference 4.23 (2.11, 7.15) 8.91 (2.65, 14.37)	<0.001 <0.001	Reference 1.86 (1.28, 4.13) 3.43 (1.87, 5.24)	<0.001 <0.001

*PPS, Peking prognostic score; NPS, Naples prognostic score; mSIS, modified systemic inflammation score; CONUT, controlling nutritional status; PNI, prognostic nutritional index; mGPS, modified Glasgow prognostic score; and BMI, body mass index.*

### Definition of Sarcopenia

According to the recent European Working Group on Sarcopenia in Older People (EWGSOP) guidelines, sarcopenia was defined as the combination of low muscle mass plus low grip strength or slow gait speed (2). Low muscle strength overtakes the role of low muscle mass as a principal determinant of sarcopenia definition (2). As the design of our study is retrospective, information about muscle function (muscle strength or physical performance) cannot be collected. Thus, we focused on muscle mass evaluation to determine patients with sarcopenia. CT was used to accurately quantify muscle mass. By adopting a public semi-automatic software [body mass index (BMI) measurement approach, version 1.0; https://sourceforge.net/projects/muscle-fat-area-measurement/], we determined the cross-section areas (CSAs) of paraspinal muscles, psoas muscles, and rectus, oblique, and transverse abdominal muscles at the third lumbar vertebra (L3) level with the threshold being -29 to 150 Hounsfield units (21, 22). The radiologist who had 5 years of experience in abdominal imaging and was blinded to subject information was invited for analyses by the de-identified Digital Imaging and Communications in Medicine files. We later normalized the L3 SMI to the patient stature below: lumbar total muscle CSA (cm^2^)/height (m^2^). In addition, the thresholds for CT-based sarcopenia of sex-specific L3 SMI were ≤34.9 cm^2^/m^2^ and ≤40.8 cm^2^/m^2^ for women and men, respectively, which was created by the Zhuang et al. for the Chinese population (23).

### Evaluation of Lymphocyte-C-Reactive Protein Ratio

Routine blood examination was conducted 1 week preoperatively, and we acquired these results in Laboratory Database of the National Cancer Center (Beijing, China). There was no sign of active infection, chronic inflammation, or pyrexia (axillary temperature ≥ 37.2°C/99.0°CF) among the enrolled patients. We divided lymphocyte count (number/ml) by CRP content (mg/dl) to determine LCR and adopted the threshold LCR utilized for CRC and GC in previous studies (14, 15). Low LCR was defined as LCR ≤ 6,000.

### Establishment of the Peking Prognostic Score

The PPS was calculated according to the preoperative sarcopenia status and LCR. All cases were classified into 4 groups. The score was 0 for patients showing no sarcopenia or LCR > 6,000, 1 for patients with sarcopenia and LCR > 6,000, 2 for those showing no sarcopenia with LCR ≤ 6,000, and 3 for those having the sarcopenia and LCR ≤ 6,000. The patients were divided into 3 groups according to the PPS value, including group 0 (PPS, 0), group 1 (PPS, 1 or 2), and group 2 (PPS, 3; Supplementary Table 1).

### Definition of Other Scoring Systems

We employed the formula below to determine PNI according to the previous description, namely, 10 × albumin content in serum (g/dl) + 0.005 × peripheral blood lymphocyte number (number/mm^2^). We later classified patients as low (PNI ≤ 45) or high (PNI > 45) PNI group (24). We determined CONUT scores in line with preoperative serum albumin content, lymphocyte number in peripheral blood, and total cholesterol (TC) level. We divided cases as low (<4) or high (≥4) CONUT score group (16). The mGPS score was 0 for cases showing CRP ≤ 1.0 mg/dl despite the albumin contents, 1 for cases showing albumin ≥ 3.5 g/dl and CRP > 1.0 mg/dl, while 2 for cases showing albumin < 3.5 g/dl and CRP > 1.0 mg/dl. The mSIS score was rated as 0 for those showing Alb content ≥ 40 g/L, 1 for those showing LMR ≥ 3.4 and Alb content < 40 g/L, while 2 for those having LMR < 3.4 and Alb content < 40 g/L (17). NPS was calculated by albumin concentration in serum, TC level, LMR, and NLR. Based on the method described by Galizia et al. in a previous study, patients were classified into three groups (20).

### Statistical Methods

We analyzed continuous and categorical data through *t*-tests and chi-square test, respectively. Survival curves were performed based on Kaplan–Meier (KM) analysis, while log-rank test was adopted for analyzing the differences between them. Upon univariate analysis, we included significant variables for multivariate analysis by using the Cox regression model. Thereafter, we adopted t-ROC curves and estimated AUC values to analyze PPS’ prognostic ability with PNI, CONUT, mSIS, mGPS, and NPS. The *P* < 0.05 (two-sided) stood for statistical significance. We employed Rver.4.0.2 (R Foundation for Statistical Computing, Vienna, Austria) and SPSS18.0 (SPSS Inc., Chicago, IL, United States) in statistical analyses. Besides, C-index was calculated by R package “rms,” whereas t-ROC curves were analyzed by R package “timeROC.”

## Results

### Patient Features

The discovery cohort enrolled altogether 774 patients with non-metastatic GC, including 639 (82.5%) men along with 135 (17.5%) women. Their mean age when the surgery was performed was 62.3 years [interquartile range (IQR): 57–70 years]. According to our thresholds, 381 (49.2%) cases had sarcopenia. Based on pTNM classification system, there were 157 (20.2%), 243 (31.5%), and 374 (48.3%) stages I, II, and III patients, respectively. Among the 774 cases, 478 (61.7%) cases accepted adjuvant chemotherapy. In line with the PPS system, 166 cases had 0 point (21.4%), 245 cases had 1 point (ratio, 31.6%), 227 cases had 2 points (ratio, 29.3%), and 136 cases had 3 points (ratio, 17.7%). The patients were divided into 3 groups. Therefore, 166 (21.4%) patients were assigned in group 0 (PPS 0), 472 (60.9%) patients were assigned in group 1 (PPS 1 or 2), and 136 (17.7%) patients were assigned in group 2 (PPS 3). The validation cohort enrolled 575 patients in our hospital from March 2016 to September 2019 (Supplementary Table 2).

### Associations Between Peking Prognostic Score System and Clinicopathological Features

Table 1 displays the associations between PPS and clinicopathological features in the discovery cohort. An increased PPS was markedly related to advanced age (≥65.0 years; *P* < 0.001) and reduced BMI (<18.5 kg/m^2^; *P* = 0.001). As for tumor factors, we found that PPS was markedly related to pTNM stage (*P* < 0.001), adjuvant chemotherapy (*P* < 0.001), perineural invasion (*P* = 0.002), and vascular invasion (*P* < 0.001). These results were identified in the validation cohort (Supplementary Table 2).

### Prognostic Impact of Peking Prognostic Score in Patients With Gastric Cancer

**FIGURE 2:**
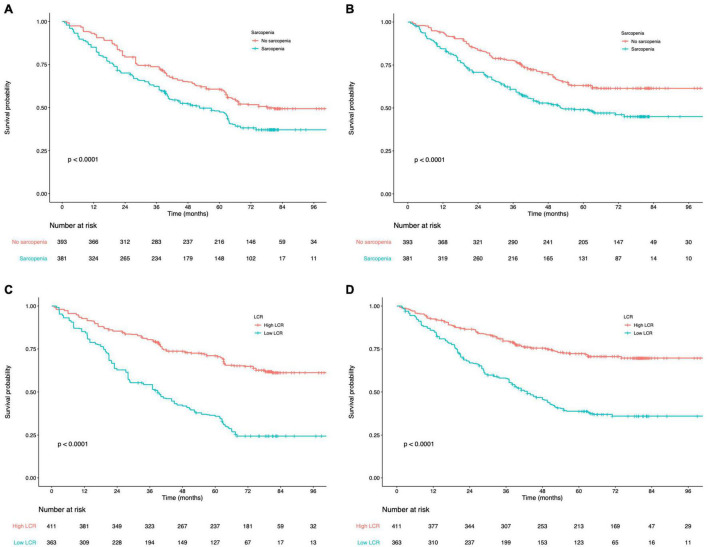
**(A)** Kaplan–Meier curves for overall survival according to the preoperative sarcopenia status. **(B)** Kaplan–Meier curves for disease-free survival according to the preoperative sarcopenia status. **(C)** Kaplan–Meier curves for overall survival according to the preoperative LCR. **(D)** Kaplan–Meier curves for disease-free survival according to the preoperative LCR. LCR, lymphocyte-to-C-reactive protein ratio.

**FIGURE 3:**
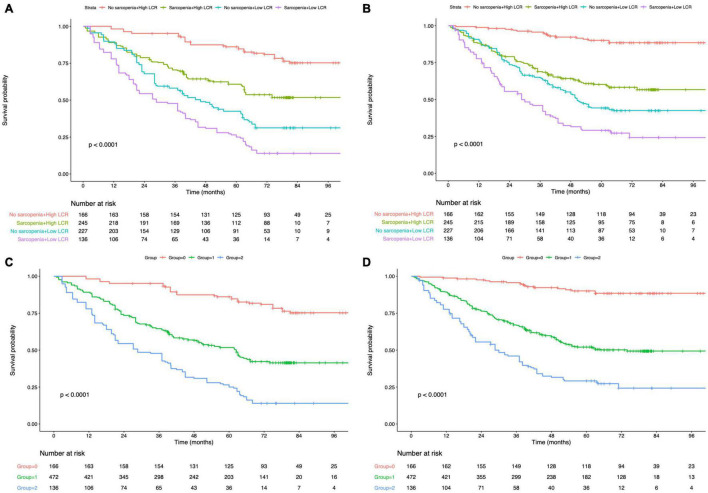
Kaplan–Meier survival analysis of overall survival according to Peking prognostic score **(A)** and group **(C)**. Kaplan–Meier survival analysis of disease-free survival according to Peking prognostic score **(B)** and group **(D)**. LCR, lymphocyte-to-C-reactive protein ratio.

**FIGURE 4:**
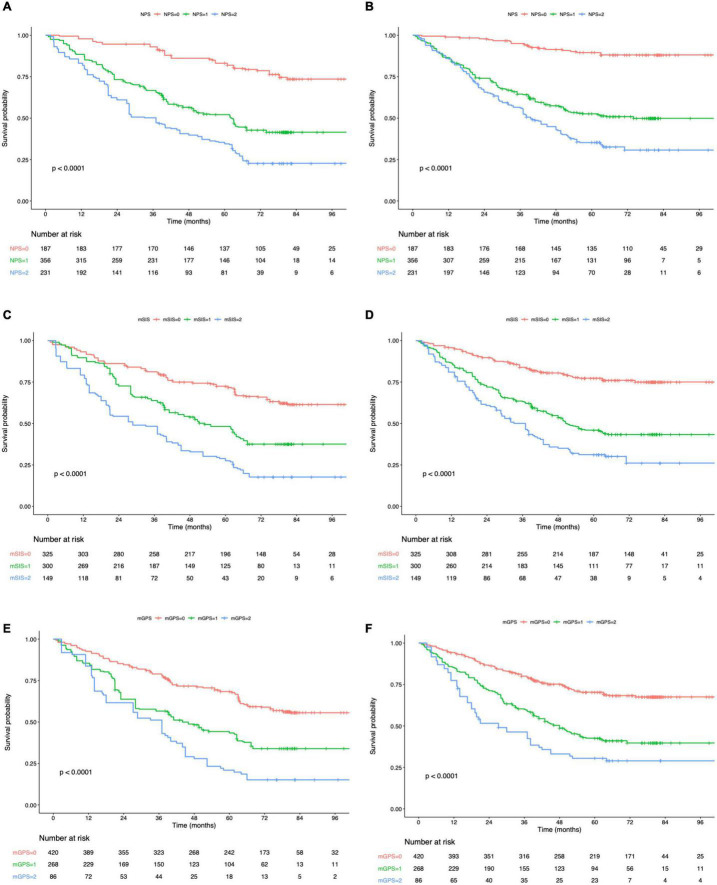
Kaplan–Meier survival analysis of overall survival **(A)** and disease-free survival **(B)** according to NPS. Kaplan–Meier survival analysis of overall survival **(C)** and disease-free survival **(D)** according to mSIS. Kaplan–Meier survival analysis of overall survival **(E)** and disease-free survival **(F)** according to mGPS. NPS, Naples prognostic score; mSIS, modified systemic inflammation score; and mGPS, modified Glasgow prognostic score.

The K-M curves displayed the significantly different OS and DFS in sarcopenia compared with non-sarcopenia groups (log-rank test, *P* < 0.001 for OS and DFS; Figures 2A,B). Moreover, as revealed by multivariate Cox regression, patients with sarcopenia had markedly inferior OS (HR = 1.77; 95% CI = 1.18–2.05, *P* < 0.001) and DFS (HR = 1.72; 95% CI = 1.16–2.28, *P* < 0.001; Tables 2, 3). As revealed by K-M survival curves stratified by LCR, low LCR predicted the dismal prognostic outcome (log-rank test, *P* < 0.001 for OS and DFS; Figures 2C,D). We discovered from multivariate analysis that LCR independently predicted OS (HR = 2.62; 95% CI = 1.72–3.38, *P* < 0.001) and DFS (HR = 2.51; 95% CI = 1.60–3.46, *P* < 0.001; Tables 2, 3). Based on the KM analysis, PPS scores of 0–3 were closely associated with survival; besides, every 1-point increase in preoperative PPS predicted dismal prognosis (OS and DFS: log-rank test, *P* < 0.001; Figures 3A,B). The KM survival analyses indicated that OS and DFS were markedly shortened with the increased PPS group in a stepwise manner (OS and DFS: log-rank test, *P* < 0.001; Figures 3C,D). The validation cohort confirmed this finding (Supplementary Figure 1). We also verified PPS to be the factor that independently predicted OS (PPS group 1: HR = 2.32, 95% CI = 1.37–4.94, *P* < 0.001; PPS group 2: HR = 4.67, 95% CI = 2.12–8.65, *P* < 0.001) together with DFS (PPS group 1: HR = 1.86, 95% CI = 1.28–4.13, *P* < 0.001; PPS group 2: HR = 3.43, 95% CI = 1.87–5.24, *P* < 0.001) upon multivariate analysis (Tables 2, 3). We further evaluated the prognostic value of PPS in the validation cohort and confirmed that preoperative PPS independently estimates patient OS and DFS (Supplementary Tables 3, 4). Figure 4 shows the OS and DFS curves for other prognostic scoring systems, including NPS, mSIS, and mGPS (Figure 4). Supplementary Figure 2 shows the OS and DFS curves for CONUT and PNI (Supplementary Figure 2). According to multivariable regression, mSIS, mGPS, and NPS estimated OS and DFS independently, whereas CONUT and PNI just independently predicted OS (Tables 2, 3). Additional factors that independently predicted prognosis included age, BMI, pTNM stage, adjuvant chemotherapy, perineural invasion, and vascular invasion (Tables 2, 3).

### Prognostic Value of Peking Prognostic Score

**FIGURE 5:**
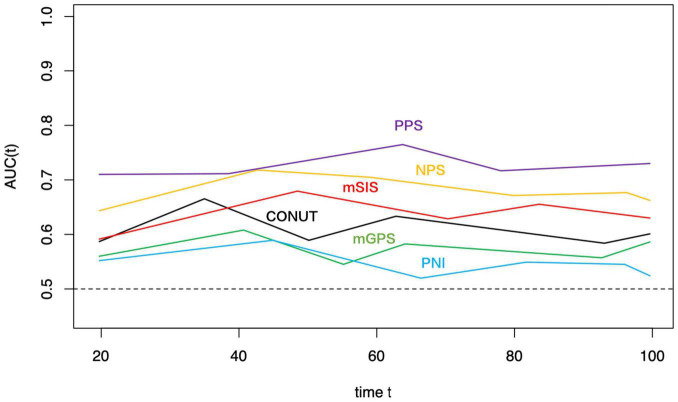
Time-dependent ROC curves of PPS, NPS, mSIS, CONUT, mGPS, and PNI for prediction of over survival. The horizontal axis represents the year after surgery, and the vertical axis represents the estimated AUC for survival at the time of interest. PPS, Peking prognostic score; NPS, Naples prognostic score; mSIS, modified systemic inflammation score; CONUT, controlling nutritional status; PNI, prognostic nutritional index; and mGPS, modified Glasgow prognostic score.

The t-ROC curve was constructed to compare PPS’ prognostic significance with PNI, CONUT, mGPS, mSIS, and NPS. Based on the t-ROC curve survival analyses using the above-mentioned scoring systems, PPS, NPS, mSIS, CONUT, mGPS, and PNI achieved the AUC values of 0.741, 0.689, 0.656, 0.627, 0.578, and 0.543, respectively, in predicting the 5-year OS (Figure 5). It showed that PPS served as the highly sensitive factor for predicting GC prognosis. In addition, PPS was markedly more accurate compared with NPS, mSIS, CONUT, mGPS, and PNI in the prediction of the 5-year DFS (Supplementary Figure 3).

## Discussion

This study explored PPS’ prognostic significance among GC cases receiving radical surgery. As a result, PPS was identified as the prognostic index for predicting long-term prognosis for the patient with GC independently. Based on our results, PPS showed close relation with OS and DFS, and cases showing an increased PPS were associated with poor OS and DFS.

In the 19th century, Virchow first reported systematically the association between inflammation and cancer. Thereafter, the host-tumor interactions-based systemic inflammation has been identified to be the 7th hallmark of malignant tumors. On this basis, more and more articles explore the possible prognostic value of systemic inflammatory biomarkers like albumin, platelets, CRP, lymphocytes, and neutrophils, as well as their combination ratios (like CAR, LCR, NLR, LMR, and PLR) as the biomarkers to predict the prognosis of various malignancies (14, 15, 25). Serum albumin content has been incorporated into many scoring systems at present. Particularly, the reduced serum albumin content indicates malnutrition and systemic inflammation, since its level decreases through some proinflammatory molecules including cytokines (26). Hyperproteinemia predicts better survival in different cancers, including GC. LMR contains monocytes and lymphocytes, while NLR includes lymphocytes and neutrophils. Lymphocytes promote tumor immune surveillance, thus suppressing tumor cell growth, migration, and invasion (27). Tumor-infiltrating lymphocytes (TILs) are related to an improvement of cancer prognostic outcome, and it is possibly associated with the suppression of angiogenesis and anticancer effect resulting from TILs (28). In this regard, lymphopenia predicts the dismal prognostic outcome of cancer cases. It has been previously suggested that monocytes in circulation facilitate cancer development and decreased immune surveillance (29). Additionally, monocytes are discovered to enhance cancer cell migration *via* the tumor-monocyte-endothelial interaction (30). Cytotoxic CD8 T-cells have an anticancer effect, which is inhibited through an increased number of neutrophils around the tumor, resulting in cancer occurrence and development (31). Moreover, for cases having an increased NLR, the cytokines derived from neutrophils [like vascular endothelial growth factor, matrix metalloproteinases, and interleukin-18] may contribute to tumor growth (32). Serum CRP has become a frequently used biomarker that reflects systemic inflammation clinically. Moreover, the high CRP content is indicated to predict the dismal prognostic outcome of patients with GC. Okugawa et al. recently reported that LCR was the marker to predict the prognosis of CRC and GC cases (14). Two recent studies found that LCR showed was the most accurate in predicting patient prognosis relative to those existing inflammatory scores like NLR, LMR, PLR, and CAR (14, 33).

An increasing number of articles are conducted to determine the relations between malnutrition/systemic inflammation and carcinogenesis, cancer development, migration, and progression. Additionally, it is verified within different tumors like EC and GC. Currently, it is necessary to identify nutritional and inflammatory markers and formulate a new prognostic scoring system. CONUT has been calculated based on serum content, total lymphocyte number, and TC content, and it is identified to be the efficient method for assessing nutritional status (34). Additionally, Kuroda et al. found CONUT as an effective method for predicting nutritional status and long-run OS among patients with GC undergoing surgical treatment (16). PNI can estimate the immune and nutritional statuses, and it has been adopted for evaluating the general condition of patients and efficiently predicting the long-run survival of patients with GC (35). The mSIS scoring system has been established based on albumin and LMR levels before surgery, which is associated with the survival of various tumors and is a reliable inflammation scoring system (17). According to Melling et al., GPS contributed to the independent prediction of long-run GC prognosis postoperatively (36). In addition, NPS, constructed using serum ALB level, TC content, NLR, and LMR, has developed as the new inflammatory prognostic scoring system. In addition, NPS showed the prognostic significance of gastrointestinal malignancies (20). Sarcopenia, which has been confirmed as the loss of function and mass of skeletal muscle, predicts the dismal nutritional status. Furthermore, it has been currently regarded as the tumor cachexia hallmark. Sarcopenia is clinically important among cancer cases, which arouses more and more interests from researchers in the last 10 years. Sarcopenia’s prognostic significance is identified within different tumors, including GC. Sarcopenia and a higher mGPS constituted by CRP were independently related to the dismal prognosis of cases having local renal cell carcinoma (37). Previous studies indicate that sarcopenia accompanied by high NLR has an inferior OS in CRC, biliary tract cancer, and patients with stage IV GC (38–40). PPS, based on LCR and sarcopenia status, well reflects both the nutritional and inflammatory statuses of patients with GC. The PPS significantly predicts the long-run prognosis and disease relapse. Besides, this study revealed the superiority of PPS over mGPS, NPS, mSIS, CONUT, and PNI in predicting GC prognosis after radical surgery.

There are some limitations in this study. First, the recent EWGSOP suggests using the presence of loss of muscle mass plus low muscle function (strength or performance) to define sarcopenia. Low muscle strength overtakes the role of low muscle mass as a principal determinant of sarcopenia definition (2). Due to the retrospective nature of our study, information about muscle function (muscle strength or physical performance) cannot be collected. Thus, we focused on muscle mass evaluation to determine patients with sarcopenia. CT was used to assess muscle mass. There are several advantages to the use of CT. CT is widely used as a routine examination and staging method for patients with gastric cancer and can accurately quantify muscle mass. The definition of sarcopenia put forward by Zhuang et al. was utilized in this study, which defined sarcopenia criteria for the Chinese population (23). The L3-SMI thresholds for diagnosing CT-based sarcopenia were 34.9 cm^2^/m^2^ and 40.8 cm^2^/m^2^ for women and men, respectively. This study showed limited generalizability to western populations, since our adopted L3 SMI thresholds showed high specificity to geographic location. Second, the effect of preoperative PPS on predicting the prognosis of GC cases was evaluated, but selection bias still existed due to the retrospective nature. However, these findings have been subsequently confirmed in the validation cohort. We only recruited cases at a single center in China, showing ethnic homogeneity. For overcoming the above limitations, more large-scale multi-center prospective studies should be conducted. Third, although we eliminated cases receiving NACT, it remains unclear whether our enrolled cases were in an identical status before blood sampling, and our results were not applicable to patients with GC receiving NACT.

## Conclusion

We concluded that PPS before surgery was the facile and effective prognostic factor for patients with GC. The PPS showed the highest sensitivity in estimating patient prognosis relative to those existing inflammatory biomarkers, as revealed by time-dependent ROC curve survival analyses. PPS contributes to accurately predicting the prognosis and assisting decision-making among GC cases. Meanwhile, PPS can be utilized as one part of prognosis stratification before surgery, and a higher PPS predicts an increased tumor recurrence risk and the necessity for customized treatment.

## Data Availability Statement

The original contributions presented in the study are included in the article/Supplementary Material, further inquiries can be directed to the corresponding author.

## Ethics Statement

The studies involving human participants were reviewed and approved by the Ethics Review Committee of National Cancer Center/Cancer Hospital, Chinese Academy of Medical Sciences and Peking Union Medical College. Written informed consent for participation was not required for this study in accordance with the national legislation and the institutional requirements.

## Author Contributions

JX conceived the study and wrote the manuscript. WK and HH searched the database, reviewed the studies, and collected the data. XS and JX performed the statistical analyses. YL, PJ, and WL performed the revision of the manuscript. YT arranged for and provided the funding for this study. All authors reviewed the manuscript and participated in its revision. YT had full access to all of the data in the study and takes responsibility for the integrity of the data and the accuracy of the data analysis.

## Conflict of Interest

The authors declare that the research was conducted in the absence of any commercial or financial relationships that could be construed as a potential conflict of interest.

## Publisher’s Note

All claims expressed in this article are solely those of the authors and do not necessarily represent those of their affiliated organizations, or those of the publisher, the editors and the reviewers. Any product that may be evaluated in this article, or claim that may be made by its manufacturer, is not guaranteed or endorsed by the publisher.
