# Supplementary material for: Peking Prognostic Score, Based on Preoperative Sarcopenia Status, Is a Novel Prognostic Factor in Patients With Gastric Cancer
Source: Front Nutr. 2022 Jun 6;9:910271. doi: 10.3389/fnut.2022.910271 (PMC9210445; doi:10.3389/fnut.2022.910271)
Supplement: Supplementary file 1 [file Data_Sheet_1.DOCX]

**Peking prognostic score, based on preoperative sarcopenia status, is a novel prognostic factor in patients with gastric cancer**

Jianping Xiong^1^, Haitao Hu^1^, Wenzhe Kang^1^, Yang Li^1^, Peng Jin^1^, Xinxin Shao^1^, Weikun Li^1^, Yantao Tian^1,†^

**^1^** Department of Pancreatic and Gastric Surgery, National Cancer Center/ National Clinical Research Center for Cancer/Cancer Hospital, Chinese Academy of Medical Sciences and Peking Union Medical College, Beijing, People’s Republic of China.

**Running title:** A novel prognostic index for gastric cancer

**Corresponding Author:** Yantao Tian^†^, Department of Pancreatic and Gastric Surgery, National Cancer Center/ National Clinical Research Center for Cancer/Cancer Hospital, Chinese Academy of Medical Sciences and Peking Union Medical College, Panjiayuan street, Chaoyang District, Beijing 100021, People’s Republic of China, Tel\Fax: +86-10-87787120, Email: tianyantao@cicams.ac.cn


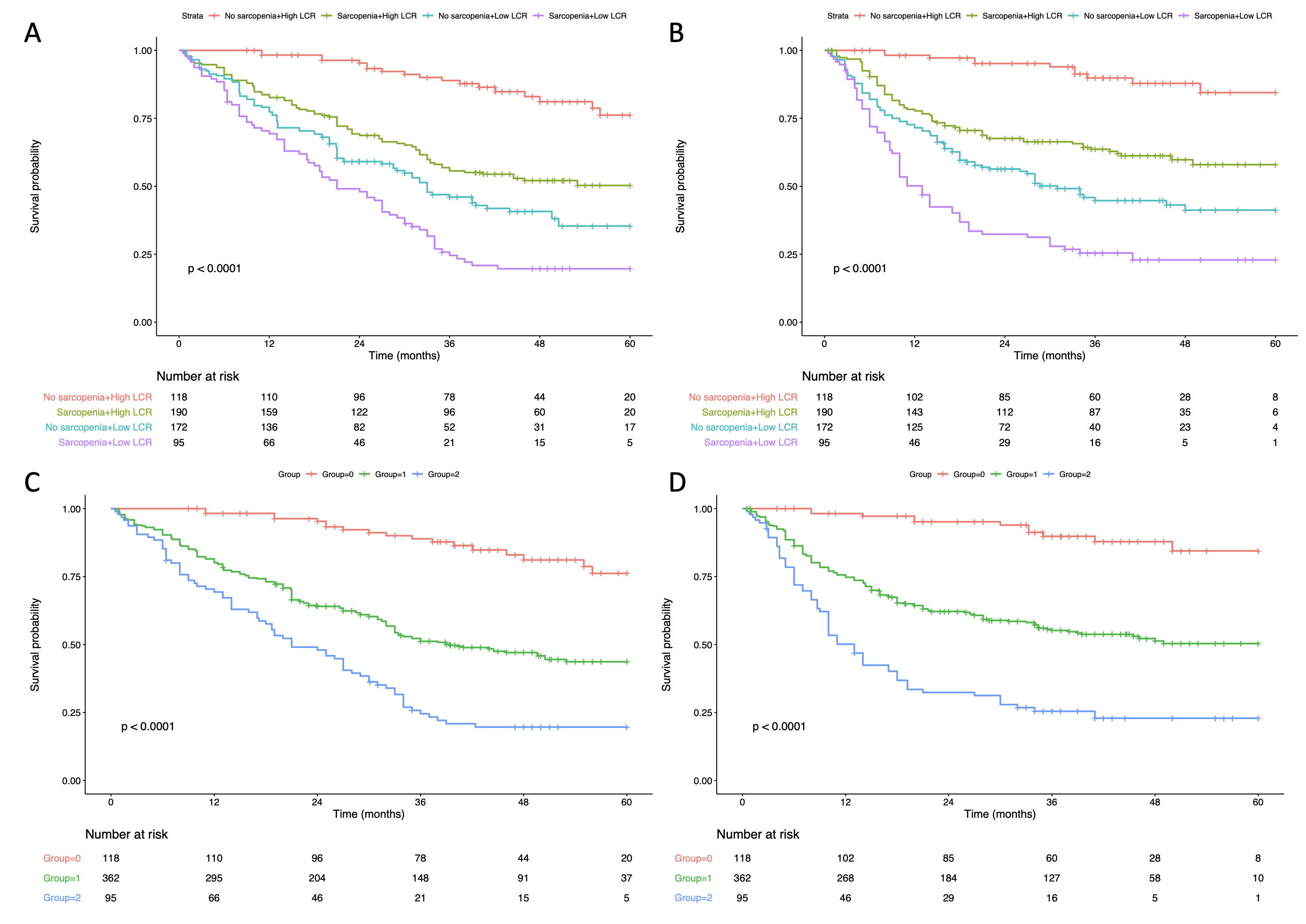


**Supplemental Figure 1.** Kaplan–Meier survival analysis of overall survival according to Peking prognostic score (A) and group (C) in the validation cohort. Kaplan–Meier survival analysis of disease-free survival according to Peking prognostic score (B) and group (D) in the validation cohort. LCR, lymphocyte-to-C-reactive protein ratio.


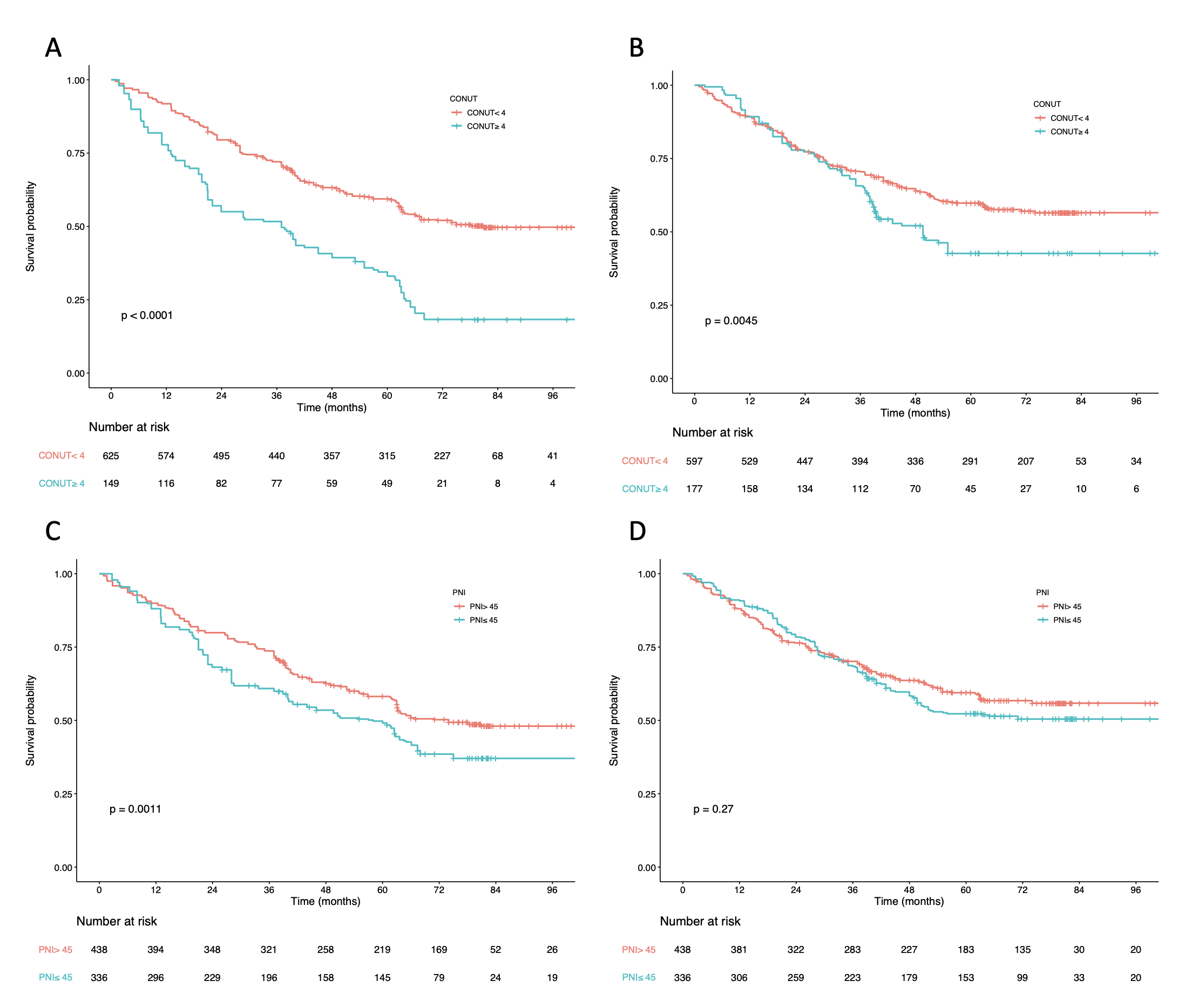


**Supplemental Figure 2.** Kaplan–Meier survival analysis of overall survival (A) and disease-free survival (B) according to CONUT. Kaplan–Meier survival analysis of overall survival (C) and disease-free survival (D) according to PNI. CONUT, controlling nutritional status; PNI, prognostic nutritional index.


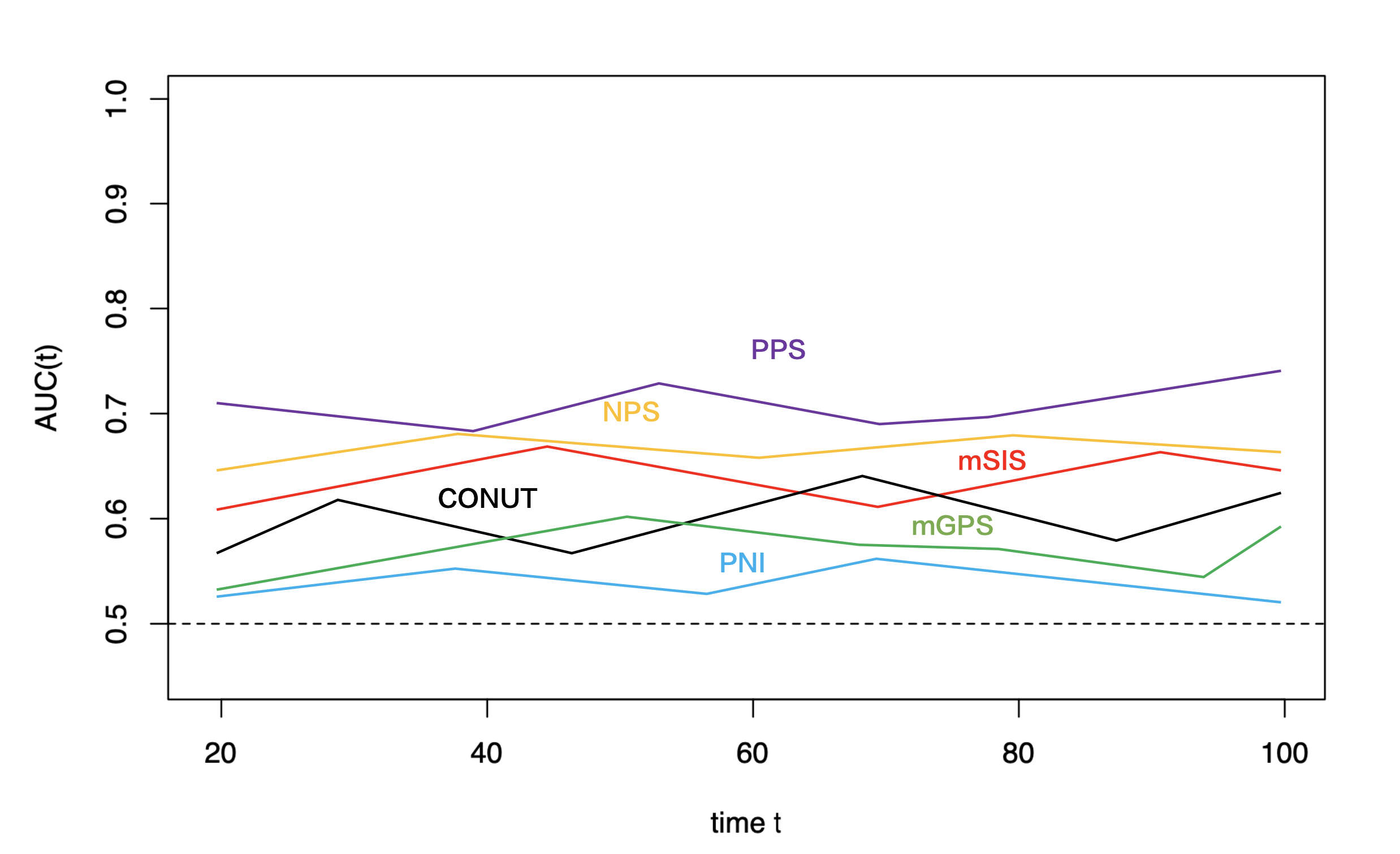


**Supplemental Figure 3.** Time-dependent ROC curves of PPS, NPS, mSIS, CONUT, mGPS and PNI for prediction of disease-free survival. The horizontal axis represents year after surgery, and the vertical axis represents the estimated AUC for survival at the time of interest. PPS, Peking prognostic score. NPS, naples prognostic score; mSIS, modified systemic inflammation score; CONUT, controlling nutritional status; PNI, prognostic nutritional index. mGPS, modified Glasgow prognostic score.

**Supplemental Table 1.** The definition of Peking prognostic score.

| Scoring System | Score | Group |
| --- | --- | --- |
| **The PPS** |  |  |
| Sarcopenia (No ) and LCR (>6000) | 0 | 0 |
| Sarcopenia (Yes) and LCR (>6000) | 1 | 1 |
| Sarcopenia (No ) and LCR (≤6000) | 2 | 1 |
| Sarcopenia (Yes ) and LCR (≤6000 ) | 3 | 2 |
|  |  |  |
|  |  |  |

LCR, lymphocyte-to-C-reactive protein ratio.

**Supplemental Table 2.** Association of Peking prognostic score and clinicopathological characteristics in patients with gastric cancer (validation cohort).

| Clinicopathological features | All cases  (n = 575) | Group 0  (n = 118) | Group 1  (n = 362) | Group 2  (n = 95) | P value |
| --- | --- | --- | --- | --- | --- |
| Age  ＜65.0  ≥65.0 | 301 (52.3)  274 (47.7) | 85 (72.1)  33 (27.9) | 178 (49.1)  185 (50.9) | 38 (36.8)  57 (63.2) | <0.001 |
| Gender  Male  Female | 439 (77.4)  136 (23.6) | 94 (79.7)  24 (20.3) | 271 (74.8)  91 (25.2) | 74 (77.9)  21 (22.1) | 0.549 |
| BMI (kg/m2)  ≥18.5  ＜18.5 | 542 (94.3)  33 (5.7) | 116 (98.4)  2 (1.6) | 343 (94.8)  19 (5.2) | 83 (87.4)  12 (12.6) | <0.001 |
| Vascular invasion  Negative  Positive | 382 (67.5)  193 (33.5) | 100 (81.2)  18 (15.3) | 238 (63.6)  124 (34.2) | 44 (46.3)  51 (53.7) | <0.001 |
| Perineural invasion  Negative  Positive | 462 (80.4)  113 (19.6) | 106 (89.8)  12 (10.2) | 293 (80.2)  69 (19.1) | 66 (69.5)  29 (30.5) | 0.001 |
| Tumor location  Upper  Middle /Lower | 190 (33.2)  385 (66.9) | 43 (36.4)  75 (63.6) | 115 (31.8)  247 (68.2) | 32 (33.7)  63 (62.3) | 0.201 |
| pTNM stage  I  II  III | 158 (27.4)  186 (32.3)  231 (40.3) | 60 (50.8)  30 (25.3)  28 (23.9) | 88 (24.3)  121 (33.4)  154 (42.3) | 10 (10.5)  35 (37.9)  49 (51.6) | <0.001 |
| Adjuvant chemotherapy  No  Yes | 212 (36.9)  363 (63.1) | 70 (59.3)  48 (40.7) | 119 (32.8)  243 (67.2) | 23 (24.3)  72 (75.7) | <0.001 |

BMI, body mass index.

**Supplemental Table 3.** Univariate and multivariate analysis of clinicopathologic variables in relation to overall survival in patients with gastric cancer (validation cohort).

| Clinicopathological features | Univariate analysis | P value | Multivariate analysis | P value |
| --- | --- | --- | --- | --- |
| Age  ＜65.0  ≥65.0 | Reference  1.37 (1.06, 3.79) | < 0.001 | Reference  1.26 (1.04, 2.23) | < 0.001 |
| Gender  Male  Female | Reference  0.79 (0.65, 2.89) | 0.235 |  |  |
| BMI (kg/m2)  ≥18.5  ＜18.5 | Reference  3.23 (1.57, 4.22) | < 0.001 | Reference  2.39 (1.38, 3.16) | < 0.001 |
| Vascular invasion  Negative  Positive | Reference  2.31 (1.46, 3.85) | < 0.001 | Reference  1.90 (1.21, 2.78) | < 0.001 |
| Perineural invasion  Negative  Positive | Reference  1.99 (1.22, 3.17) | < 0.001 | Reference  1.52 (1.16, 2.33) | 0.008 |
| Tumor location  Upper  Middle /Lower | Reference  0.83 (0.57, 2.70) | 0.301 |  |  |
| pTNM stage  I  II  III | Reference  2.42 (1.44, 5.89)  9.64 (3.03, 18.13) | < 0.001  < 0.001 | Reference  1.93 (1.10, 3.06)  6.51 (3.09, 9.75) | < 0.001  < 0.001 |
| Adjuvant chemotherapy  Yes  No | Reference  2.49 (1.40, 5.64) | <0.001 | Reference  1.71 (1.35, 2.88) | < 0.001 |
| Sarcopenia  without  with | Reference  3.48 (1.53, 5.28) | <0.001 | Reference  1.93 (1.21, 2.64) | <0.001 |
| Lymphocyte: C-reactive protein ratio  ＞6000  ≤6000 | Reference  4.01 (2.14, 5.47) | <0.001 | Reference  2.67 (1.64, 3.85) | <0.001 |
| PNI  ＞45  ≤45 | Reference  3.35 (1.39, 6.06) | < 0.001 | Reference  1.82 (1.20, 3.14) | <0.001 |
| CONUT  ＜4  ≥4 | Reference  4.27 (1.92, 6.31) | < 0.001 | Reference  2.49 (1.37, 3.99) | <0.001 |
| mSIS  0  1  2 | Reference  2.44 (1.26, 6.18)  4.91 (2.02, 9.35) | < 0.001  < 0.001 | Reference  1.54 (1.23, 3.69)  2.16 (1.40, 4.27) | < 0.001  < 0.001 |
| mGPS  0  1  2 | Reference  3.25 (2.21, 6.83)  5.04 (2.40, 9.83) | < 0.001  < 0.001 | Reference  2.27 (1.33, 3.46)  3.23 (1.94, 5.85) | <0.001  <0.001 |
| NPS  0  1  2 | Reference  2.86 (1.42, 5.74)  4.97 (2.63, 9.20) | < 0.001  < 0.001 | Reference  2.27 (1.23, 3.74)  3.35 (1.61, 5.58) | < 0.001  < 0.001 |
| PPS  0  1  2 | Reference  4.66 (2.59, 7.91)  9.25 (2.68, 18.03) | < 0.001  < 0.001 | Reference  2.41 (1.40, 4.92)  4.53 (2.15, 9.27) | < 0.001  < 0.001 |

PPS, Peking prognostic score. NPS, naples prognostic score. mSIS, modified systemic inflammation score. CONUT, controlling nutritional status. PNI, prognostic nutritional index. mGPS, modified Glasgow prognostic score. BMI, body mass index.

**Supplemental Table 4.** Univariate and multivariate analysis of clinicopathologic variables in relation to disease-free survival in patients with gastric cancer (validation cohort).

| Clinicopathological features | Univariate analysis | P value | Multivariate analysis | P value |
| --- | --- | --- | --- | --- |
| Age  ＜65.0  ≥65.0 | Reference  1.58 (1.10, 2.67) | < 0.001 | Reference  1.27 (1.05, 1.88) | < 0.001 |
| Gender  Male  Female | Reference  0.82 (0.63, 2.74) | 0.372 |  |  |
| BMI (kg/m2)  ≥18.5  ＜18.5 | Reference  2.95 (1.43, 4.91) | < 0.001 | Reference  2.12 (1.24, 3.05) | < 0.001 |
| Vascular invasion  Negative  Positive | Reference  2.26 (1.29, 4.83) | < 0.001 | Reference  1.63 (1.14, 2.26) | < 0.001 |
| Perineural invasion  Negative  Positive | Reference  1.87 (1.21, 3.72) | < 0.001 | Reference  1.55 (1.12, 2.48) | 0.005 |
| Tumor location  Upper  Middle /Lower | Reference  0.74 (0.52, 2.80) | 0.607 |  |  |
| pTNM stage  I  II  III | Reference  3.24 (1.75, 6.31)  9.10 (3.31, 17.64) | < 0.001  < 0.001 | Reference  1.79 (1.14, 2.74)  4.68 (2.42, 8.19) | < 0.001  0.001 |
| Adjuvant chemotherapy  Yes  No | Reference  3.88 (1.64, 5.03) | < 0.001 | Reference  1.71 (1.30, 2.89) | < 0.001 |
| Sarcopenia  without  with | Reference  3.02 (1.63, 5.60) | <0.001 | Reference  1.67 (1.11, 2.75) | <0.001 |
| Lymphocyte: C-reactive protein ratio  ＞6000  ≤6000 | Reference  4.56 (2.32, 6.67) | <0.001 | Reference  2.39 (1.53, 3.74) | <0.001 |
| PNI  ＞45  ≤45 | Reference  3.16 (2.05, 7.31) | 0.002 | Reference  1.92 (0.89, 2.62) | 0.150 |
| CONUT  ＜4  ≥4 | Reference  3.50 (1.95, 6.17) | <0.001 | Reference  2.26(0.67, 3.73) | 0.276 |
| mSIS  0  1  2 | Reference  2.39 (1.26, 5.16)  4.51 (2.38, 8.42) | < 0.001  < 0.001 | Reference  1.57 (1.21, 3.08)  2.02 (1.50, 4.16) | < 0.001  < 0.001 |
| mGPS  0  1  2 | Reference  3.13 (1.84, 6.02)  4.43 (2.01, 7.76) | < 0.001  < 0.001 | Reference  2.01 (1.37, 3.90)  2.65 (1.32, 5.73) | < 0.001  < 0.001 |
| NPS  0  1  2 | Reference  2.77 (1.56, 6.35)  5.01 (2.79, 9.83) | < 0.001  < 0.001 | Reference  2.08 (1.20, 3.74)  3.20 (1.55, 5.82) | < 0.001  < 0.001 |
| PPS  0  1  2 | Reference  4.41 (2.19, 8.24)  8.62 (2.80, 15.61) | < 0.001  < 0.001 | Reference  1.93 (1.25, 4.66)  3.65 (1.93, 6.04) | < 0.001  < 0.001 |

PPS, Peking prognostic score. NPS, naples prognostic score. mSIS, modified systemic inflammation score. CONUT, controlling nutritional status. PNI, prognostic nutritional index. mGPS, modified Glasgow prognostic score. BMI, body mass index.
